# Impact of self‐perception of aging on mortality of older patients in oncology

**DOI:** 10.1002/cam4.2819

**Published:** 2020-02-04

**Authors:** Sarah Schroyen, Luc Letenneur, Pierre Missotten, Guy Jérusalem, Stéphane Adam

**Affiliations:** ^1^ Psychology of Aging Unit University of Liège Liège Belgium; ^2^ UMR1219 Bordeaux Population Health INSERM University of Bordeaux Bordeaux France; ^3^ Laboratory of Medical Oncology University of Liège Liège Belgium; ^4^ Department of Medical Oncology CHU Sart‐Tilman Liège Liège Belgium

**Keywords:** cancer risk factors, medical oncology, psychosocial studies

## Abstract

**Objective:**

Several studies show that self‐perception of aging (SPA) is a significant predictor of mental and physical health. In this study, we analyze the effect of SPA on mortality in the specific context of geriatric oncology.

**Methods:**

The sample constituted of 140 individuals aged 65 years and older suffering from a recent nonmetastatic cancer (breast, lung, gynecological, or hematological), followed up to 6 years. We used Cox proportional hazards model to assess the effect of SPA at baseline on mortality. It was adjusted for age, gender, educational and cognitive level, oncological information (the site and kind of cancer), number of comorbidities, and physical and mental health at baseline.

**Results:**

Patients were aged 73 years at diagnosis and were more often women (85.7%). Individuals with more negative SPA were 3.62 times more likely to die than those with a more positive SPA, with control of gender, age, education and cognitive level, mental and physical health, the category (breast, lung, gynecological, or hematological), and kind (initial or recurrence) of cancer.

**Conclusions:**

These findings suggest that SPA influence the mortality of older people in the particular context of oncology. Therefore, the need to change our attitudes toward aging and older people implied indirectly by these results is discussed.

## Graphical abstract

This research supports the view that self‐perception of aging (SPA) could be considered as a risk factor of vulnerability in oncology. Indeed, we have followed 140 patients with a 6‐year follow‐up and our results showed that individuals with more negative SPA are 3.66 times more likely to die than those with a more positive SPA. This result remains after controlling for gender, age, education and cognitive level, mental and physical health, the category (breast, lung, gynecological, or hematological), and kind (initial or recurrence) of cancer.

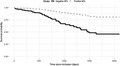

## 1 BACKGROUND

Several longitudinal studies show that self‐perception of aging (SPA) is a significant predictor of how healthy and how long people live.^1^, ^2^ For example, illustrating the impact of SPA on mental health, a research involving 385 individuals over 38 years observed that participants with more negative initial SPA had a 30.2% greater decline in memory performance than participants with less negative age stereotypes.^3^ Moreover, the impact on cognitive functioning is shown in a 4‐year study, observing that people with negative age beliefs are more likely to develop dementia than those with positive ones.^4^ Similar results were observed for physical health: indeed, in a research lasting 18 years, it was demonstrated that participants with a more negative SPA at baseline reported worse functional health during follow‐ups in comparison with those with a more positive SPA.^5^ Such results were also observed when objective cardiovascular events (angina attacks, strokes, etc) were used as outcomes: within a 38‐year follow‐up, 25% of participants in the negative age stereotypes group experienced a cardiovascular event, in comparison to 13% in the positive age stereotypes group.^6^ In addition to these findings, researches also demonstrate that SPA is associated with mortality. Indeed, the Berlin Aging Study analyzed the impact of 17 indicators of psychological functioning (personality, subjective well‐being…) on mortality: with a 6‐year follow‐up, one of the strongest psychological predictor of mortality is dissatisfaction with aging (risk ratio of 1.36). The effect remains significant after controlling age, socio‐demographic characteristics, health measures, and 16 other psychological variables (as dissatisfaction with life, loneliness…).^7^ These results were confirmed by a 23‐year study, showing that older individuals with an initial more negative SPA lived 7.5 years fewer than those with a more positive SPA.^8^ Recently, a study confirms such impact of SPA on mortality even in very old participants (over 78 years) in China.^9^

Such relationships between SPA and mortality could be surprising at first sight: how can we explain that the perception I have about my own aging will impact how long I live? The *stereotype embodiment theory* provides some hypotheses about this link. It explains that people internalize age stereotypes all along their life. Once individuals become older, these stereotypes become self‐relevant and so, contribute to the formation of their SPA, which can have physiological consequences.^10^ According to this theory, there are three possible pathways to explain this mechanism: physiological, behavioral, and psychological processes. Yet, through the physiological pathway, it hypothesized that negative SPA increases the stress level and, in turn, it produces negative health consequences. Supporting this explanation, a study has found that older people with a more positive SPA have a lower cortisol level (which is a primary stress biomarker).^11^ The behavioral pathways assume that people with a more positive SPA are more involved in positive health behavior. This has been shown in several studies.^12^, ^13^, ^14^ Indeed, people with a more negative SPA have less preventive health behaviors (such a balance diet, exercising, minimizing alcohol, or tobacco consumption) over the next two decades.^12^ In consequences, people with more positive SPA are less likely to become obese.^15^ Also, more negative SPA is associated with a higher likelihood of health care delay and more perceived barriers to care.^13^ Finally, the psychological way is based on the self‐fulfilling prophecy: if people expect to fail a task, it will negatively affect their performance, which could reinforce their negative expectations of failure (and so create a vicious circle). By this way, people exposed to negative age stereotypes (eg, Alzheimer's, decline, senile, etc) have worse memory performance.^16^ Moreover, individuals who had been exposed to negative aging stereotypes had a weaker will to live.^17^, ^18^

All these previous longitudinal researches are done in the context of normal aging. However, few studies have analyzed the impact of SPA on health within a population affected by a disease. Among older people, an important health issue concerns oncology: indeed, it is estimated that by 2035, patients over 65 years of age will represent 60% of newly diagnosed cancers in the world.^19^ Therefore, studying SPA (and its effects) in this particular population is of great importance: anticipating older cancer patients with higher risks of more negative outcomes is a major issue and could lead to improve medical effectiveness. It is all the more important as we know that SPA is more negative in oncological context: indeed, a recent study among 1140 participants has observed that individuals with cancer have a more negative SPA than people without cancer.^20^ They also observe that a more negative SPA is associated with more negative mental and physical health even among individuals with cancer. This last observation was also observed in a previous study: among 101 patients suffering from a cancer (breast, gynecological, lung, or hematological), it was observed that patients with a more negative SPA reported worse physical and mental health.^21^ Moreover, this negative association between SPA and physical and mental health is confirmed with a 1‐year follow‐up among the same patients.^22^ However, to the best of our knowledge, the impact of SPA on mortality in geriatric oncology has never been studied. Therefore, our research aims to answer this question with a maximum of a 6‐year follow‐up.

## 2 METHODS

### 2.1 Participants

About 140 patients (mean [*M*] age = 72.96; standard‐deviation [SD] = 6.02) participated in this study, thanks to a collaboration between the department of medical oncology of the CHU Sart‐Tilman Liège University Hospital (Belgium) and the Psychology of aging unit of the University of Liège. This study was approved by the local Ethics Committees (University Teaching Hospital of Liège, Faculty of Psychology of the University of Liège: protocol #2013/86, B707201317034) and written informed consent was obtained from the patients. Eligible patients were those over 65 years old with a sufficient knowledge of French, recently diagnosed with nonmetastatic cancer (breast, lung, gynecological, or hematological cancer), without comorbid diagnosis of dementia, and with a planned treatment (ie, surgery, chemotherapy, radiotherapy, or endocrine therapy). We included patients with a newly diagnosed cancer or relapse (this parameter was controlled in the analyses). All patients were met in the (day) hospital as soon as possible after the diagnosis (mean time = 43.01 days): the inclusion began in January 2013 and ceased in December 2017. Therefore, patients have a minimum follow‐up of 1 year and a maximum of 6 years (*M* follow‐up = 33.47 months, SD = 20.19 months).

### 2.2 Materials

#### 2.2.1 Independent variable: SPA

The SPA was measured with the attitudes to aging questionnaire,^23^ translated and validated in French.^24^ This scale was specifically developed to flexibly and comprehensively assess attitudes toward the aging process as a personal experience from the perspective of older adults. For each of the 24 items of the scale (Cronbach's *α* = 0.77, which is an acceptable value^25^), participants respond on a 5‐point Likert‐type scale ranging from 1 (*strongly disagree/not at all true*) to 5 (*strongly agree/extremely true*). Although this scale can be divided into three subscales (Psychosocial loss, Physical change, and Psychological growth), we only used the total score (range: 24‐120). A higher total score reflects more positive SPA. After examination of the linearity, we decided to create two groups (first group = positive SPA, second group = negative SPA) based on the median score (87).

#### 2.2.2 Covariates

- *Demographics and medical information.* Data were collected on age, sex, and educational level at baseline. Medical information, such as the site, kind (initial or recurrent), and number of comorbidities was obtained through medical records at baseline.
- *Cognitive level* was assessed only at the baseline with the French version of the mini mental state examination.^26^ This test measures orientation, learning, attention, memory, language, and constructive praxis.
- *European Organization for Research and Treatment of Cancer Quality of Life Questionnaire Core 30 *(*EORTC QLQ‐C30*).^27^ In agreement with Giesinger et al,^28^ we excluded from data analyses one item measuring financial difficulties and two items measuring the quality of life. Based on the 27 remaining items, the questionnaire includes five functioning scales: (a) physical; (b) role; (c) emotional; (d) cognitive; and (e) social. It also measures symptomatology with three scales (Nausea and Vomiting, Fatigue, Pain) as well as with five separate items (Dyspnea, Insomnia, Appetite Loss, Constipation, and Diarrhea). All scores are transformed into a 0‐100 scale, with a higher score representing a better health. For conceptual matters, we have distinguished physical and mental health as we have done for the cross‐sectional and longitudinal study.^21^, ^22^ For physical health (Cronbach's *α* = 0.90), we have included the following parameters (19 items): (a) the physical and role functioning scales; (b) symptom scales and single items. For mental health (Cronbach's *α* = 0.78), we have included the emotional, social, and cognitive functioning scale (8 items). We created two groups for physical and mental health based on the linearity of the effect on mortality (first four quintiles vs last quintile, representing a better physical and mental health).

#### 2.2.3 Outcome: Mortality

We used the number of days from the inclusion visit to participants' death or the end of the study. The time period ranged from the baseline in January 2013 to 31 December 2018, the cutoff date for mortality data. To determine if and when patients died, we used three sources of information: (a) medical records; (b) obituary page; and (c) health insurance. First of all, we look in medical records: if the patient is still followed at the hospital (last appointment in October 2018 and at least one appointment planned) and was not indicated as dead, we considered him as alive (n = 76). If we cannot determine if the patient is dead or alive through medical records (n = 30), we look at an obituary page (http://www.enaos.net): seven patients were considered dead (match with the first and last name and date of birth). If the patient was not found on this page, we called their health insurance. By this way, four patients were considered dead (match with their insurance number).

### 2.3 Data analyses

Data analyses were performed using R statistical software and statistical significance was fixed at *P* < .05. Descriptive analyses were first conducted to report sample characteristics. Then, we conducted a Kaplan‐Meier survival analysis and compared the survival in the two SPA groups with the log‐rank test. Afterward, an univariate Cox regression to analyze the influence of self‐perceptions of aging on mortality was performed. We finished by adjusting the Cox proportional‐hazards model for age at baseline, gender, educational and cognitive level, oncological information (the site and kind of cancer), number of comorbidities, and physical and mental health at baseline. The data that support the findings of this study are available from the corresponding author upon reasonable request.

## 3 RESULTS

### 3.1 Sample characteristics

Patients' characteristics are presented in Table 1. The mean age of the 140 patients is 72.96 years and the majority (85.7%) are women. Most patients had a low level of education (79.3%). Self‐perception of aging score ranged from 45 to 114 and the median score was 87. The most common cancer is breast cancer (50.7% of participants).

**Table 1:** Descriptive characteristics of the sample (N = 140)

Characteristics	M (SD)	N (%)
Female gender		120 (85.7)
Age (y)	72.96 (6.02)	
Low education level (<high school)		111 (79.3)
Primary cancer site		
Breast		71 (50.7)
Gynecology		27 (19.3)
Lung		34 (24.3)
Hematology		8 (5.7)
Initial cancer (vs recurrence or progressive)		122 (87.1)
Comorbidities		
0		28 (20)
1 or 2		81 (57.9)
≥3		31 (22.1)
Cognitive functioning (0‐30)	27.82 (1.71)	
Good mental health		25 (17.9)
Good physical health		32 (22.7)
Positive SPA		74 (52.9)
Death		45 (32.1)

Abbreviation: SPA, self‐perception of aging.

### 3.2 Kaplan‐Meier analysis

**Figure 1:**
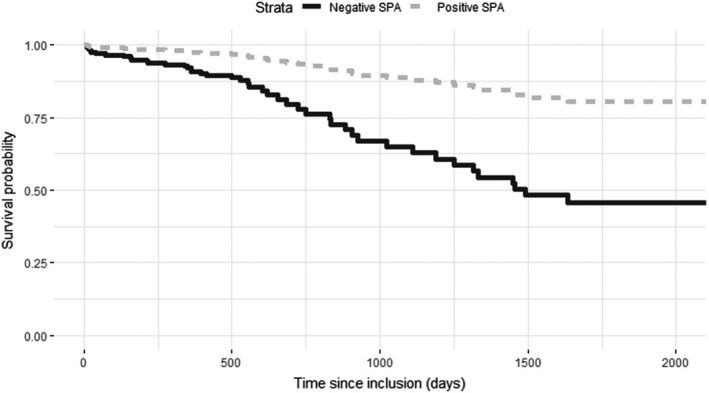
Survival probability according to self‐perception of aging (SPA), groups divided according to the median score (Kaplan‐Meier survival plot)

Among the 45 subjects who died during the follow‐up, 33 (73.33%) had a negative SPA. Better survival was observed among patients showing a positive SPA. The median survival time of those with the negative SPA was 1330 days (minimum = 1020 days, maximum = 1640 days), whereas the estimated median survival was not reached for the positive SPA. Using the log‐rang test, we found that the group of patients with a positive SPA was significantly different from the group with negative SPA (*P* < .001; see Figure 1).

### 3.3 Cox regression models

Results of the univariate Cox regression indicate that patients with a negative SPA are 3.66 times more likely to die than subjects with positive SPA (*P* < .001). After controlling for all covariates, results still show that a negative SPA increases the risk of death by 3.62 (Table 2). We can also observe significant effects of medical characteristics: lung cancer in comparison to breast cancer and having three or more comorbidities (in comparison to none) are associated with a higher likelihood of death. Also, a higher mortality is observed among men and patients who report more physical difficulties at baseline. The assumption of proportional hazards was respected by the data (*P* = .21).

**Table 2:** Results of the multivariate Cox regression (N = 140)

Characteristics	Hazard ratios	CI 95%	*P* value
SPA^a^	3.62	1.72‐7.62	<.001
Gender^b^	2.45	1.00‐5.99	.049
Age (y)	1.01	0.95‐1.08	.74
Education level^c^	0.85	0.34‐2.16	.74
Primary cancer site^d^			
Lung	8.72	3.16‐24.12	<.001
Gynecology	2.01	0.81‐4.93	.13
Hematology	1.54	0.27‐8.68	.62
Kind of cancer^e^			
Initial cancer	0.70	0.25‐1.97	.50
Comorbidities^f^			
1 or 2	2.35	0.76‐7.21	.13
≥3	4.34	1.35‐13.96	.014
Cognitive functioning	0.91	0.72‐1.15	.49
Mental health^g^	0.49	0.19‐1.28	.15
Physical heath^h^	1.69	1.81‐16.4	.002

Abbreviation: SPA, self‐perception of aging.

a 0 = Positive SPA, 1 = Negative SPA.

b 0 = female gender, 1 = male gender.

c 0 = <High School, 1 = ≥School.

d Reference = breast cancer.

e 0 = Initial cancer, 1 = Recurrence or progressive.

f 0 = no comorbidity, 1 = 1 or 2 and ≥3 comorbidities.

g 0 = Positive mental health, 1 = Negative mental health.

h 0 = Positive physical health, 1 = Negative physical health.

## 4 DISCUSSION

A lot of studies are interested in identifying factors that could influence the longevity of people. Classically, these factors are biomedical but more often, researchers are interested in psychosocial factors. For example, the subjective age (measured by asking “Many people feel older or younger than they actually are. What age do you feel?”) has been regarded as a factor of vulnerability to aging and its impact on mortality in normal aging has already been observed.^29^ Another factor largely studied is the SPA: indeed, in a nonpathological context, previous researches have demonstrated that SPA is a significant indicator of physical and mental health of older people as well as their probability of mortality.^1^, ^2^ Therefore, the aim of this study was to analyze the impact of SPA in a specific clinical context as oncology, given that cancer particularly affects the older population.^30^

Our findings confirm our hypotheses, suggesting that SPA has an impact on mortality even in this specific population: older people with a negative SPA, measured up to 6 years earlier, are 3.66 times more likely to die than people with a positive SPA. This result remains unchanged after controlling for age, gender, educational and cognitive level, oncological information (the site and kind of cancer), number of comorbidities, and physical and mental health at baseline. To the best of our knowledge, this is the first time that the impact of SPA on mortality was demonstrated in the oncological context. This impact has been observed previously in only one another specific pathological context that is respiratory illnesses.^31^

### 4.1 Clinical implications

Our research support the view that SPA could be considered as a risk factor of vulnerability.^22^ Therefore, it could constitute an interesting tool for oncologists in clinical practice, in order to assess the patient in a global way, improving predictions of possible evolution, provide an appropriate follow‐up, and so improve quality of care. Furthermore, it is important to sensitize health care professionals to the consequences of a negative SPA for older patients and so the importance of their attitudes. Indeed, it has been shown that people with negative SPA are, for example, more sensitive to patronizing communication^32^ and such communication can lead to restiveness to care.^33^ Therefore, we can make the hypothesis that it can also influence, in consequences, their mortality. For these reasons, it is necessary to be very cautious not to reinforce ageist stereotypes among older patients.

### 4.2 Study limitations and future studies

In our study, we do not have information concerning the cause of the death: the mortality of our patients could be due to cancer but also other diseases or accidents. Furthermore, we only have information concerning SPA and other covariables at the time of the diagnosis. Having information on SPA before the diagnosis and see if there is a change in SPA after it may provide a better understanding of its mechanism (for example, SPA, is it influenced by the diagnosis of a disease? And if there is an influence, is it the same for people with a negative or positive SPA before the disease?).

At last, SPA could be malleable and could potentially be modified by different interventions: therefore, future studies will need to analyze the effect of psychological intervention on SPA. Indeed, experimental studies have already demonstrated the efficiency of activation of positive aging stereotypes on older people's physical and psychological health: for instance, people have better cardiovascular measures after subliminal activation of positive aging stereotypes.^34^ Moreover, it was demonstrated that the activation of positive subliminal aging stereotypes leads to a better SPA that improved physical function.^35^ Nevertheless, to our knowledge, such intervention has never been set up yet in oncology. In this context, future studies could analyze the benefits of different procedure to change SPA among older patients in oncology, for example, by using self‐affirmation technique. This procedure aims to get the person to focus on an important aspect of his or her life (other than aging) that highlights some important value for him or her,^36^ and so will allow to reduce the negative effect of SPA.

### 4.3 Conclusion

It is certain that these results need to be replicated and further developed. Nevertheless, it suggests that even in a pathological context as oncology, negative SPA could probably enhance the likelihood of mortality. As Levy and her collaborators noted in their research,^8^ if a virus was found to have such impact on mortality, considerable effort would be set up to find a remedy: in this case, the virus is probably the stigmatization of older people in our modern society (leading to have negative SPA when we become older). Therefore, there is a need to change our attitude toward aging and older people: such changes need to be supported by policy and public health measures. For instance, it would be important to include more positive images of aging in public media or have more initiatives of intergenerational contact.^2^ By such action, we could hope that aging will be seen as a period of life not constituted on health issues and loneliness but on meaningful activities, personal growth, and pleasure.

## Data Availability

The data that support the findings of this study are available from the corresponding author upon reasonable request.
